# A landscape assessment of the activities and capacities of evidence-to-policy intermediaries (EPI) in behavioral health

**DOI:** 10.1186/s43058-023-00432-4

**Published:** 2023-05-22

**Authors:** Lars Almquist, Sarah Cusworth Walker, Jonathan Purtle

**Affiliations:** 1 Department of Health Systems and Population Health, University of Washington, Seattle, USA; 2 Department of Psychiatry and Behavioral Sciences, University of Washington, Seattle, USA; 3 Department of Public Health Policy and Management, New York University, New York City, USA

**Keywords:** Policy, Research use, Intermediaries, Behavioral health, Readiness

## Abstract

**Background:**

A significant gap exists between the production of research evidence and its use in behavioral health policymaking. Organizations providing consulting and support activities for improving policy represent a promising source for strengthening the infrastructure to address this gap. Understanding the characteristics and activities of these evidence-to-policy intermediary (EPI) organizations can inform the development of capacity-building activities, leading to strengthened evidence-to-policy infrastructure and more widespread evidence-based policymaking.

**Methods:**

Online surveys were sent to 51 organizations from English-speaking countries involved in evidence-to-policy activities in behavioral health. The survey was grounded in a rapid evidence review of the academic literature regarding strategies used to influence research use in policymaking. The review identified 17 strategies, which were classified into four activity categories. We administered the surveys via Qualtrics and calculated the descriptive statistics, scales, and internal consistency statistics using R.

**Results:**

A total of 31 individuals completed the surveys from 27 organizations (53% response rate) in four English-speaking countries. EPIs were evenly split between university (49%) and non-university (51%) settings. Nearly all EPIs conducted direct program support (mean = 4.19/5 [sd = 1.25]) and knowledge-building (4.03 [1.17]) activities. However, engagement with traditionally marginalized and non-traditional partners (2.84 [1.39]) and development of evidence reviews using formal critical appraisal methods (2.81 [1.70]) were uncommon. EPIs tend to be specialized, focusing on a group of highly related strategies rather than incorporating multiple evidence-to-policy strategies in their portfolios. Inter-item consistency was moderate to high, with scale *α*’s ranging from 0.67 to 0.85. Ratings of respondents’ willingness to pay for training in one of three evidence dissemination strategies revealed high interest in program and policy design.

**Conclusions:**

Our results suggest that evidence-to-policy strategies are frequently used by existing EPIs; however, organizations tend to specialize rather than engage in a breadth of strategies. Furthermore, few organizations reported consistently engaging with non-traditional or community partners. Focusing on building capacity for a network of new and existing EPIs could be a promising strategy for growing the infrastructure needed for evidence-informed behavioral health policymaking.

**Supplementary Information:**

The online version contains supplementary material available at 10.1186/s43058-023-00432-4.

Contributions to the literature

- A gap exists between the creation of research evidence and the use of that evidence in behavioral health policymaking. A number of organizations exist that could be well-suited to translate this evidence for decision-makers.
- Evidence-to-policy intermediaries (EPIs) are using evidence translation strategies found in the implementation science literature and are interested in learning about additional strategies.
- EPIs tend to specialize within limited subsets of strategies and may thus be missing opportunities to maximize translational impact.
- Existing EPIs report low engagement with community partners.

## Background

The integration of evidence into health-related policymaking is a stated priority for multiple national and local governments and an articulated goal of researchers working to narrow the “evidence-to-practice gap.” A number of centers, collaboratives, and initiatives have emerged over the last decade with the aim of supporting evidence-informed policymaking, including the World Health Organization-supported European Advisory Committee on Health Research [1], Health Evidence Network, and Evidence-informed Policy Network (EVIPNet); the U.S. Commission on Evidence-Based Policymaking [2]; and similar efforts in Norway [3] and Anglophone countries [4–6]. Papers have outlined research agendas to expand the field of implementation science (IS) into the policy realm [7–10]. Some concerns about applying traditional implementation frameworks to health policymaking, raised by those inside and outside of IS, focus on the irreducible complexity and non-linearity of policymaking [11] and the prohibitive costs of implementing behavioral health innovations with the infrastructure required to maintain fidelity [12]. Translation is needed between evidence production and its use in policymaking [13], but there is very little discussion of the types of organizations already positioned to play this role in local, state, and national governments.

In this paper, we investigate the distinct role that evidence-to-policy intermediaries (EPI) play compared to other types of intermediary organizations focused on mental and substance use services (hereafter termed behavioral health) as described in the field [14, 15] particularly in the strategies used to narrow the evidence-to-policy gap while ensuring the application of evidence is responsive to local political conditions. We present cross-sectional data from a survey of 40 behavioral health EPIs to provide the field with a snapshot of these organizations, including translational techniques currently in use, sources of funding and financial stability, and the perceived value of IS to their work.

A substantial gap remains between the production of research evidence and the inclusion of evidence in policymaking. This gap is particularly acute with regard to behavioral health research [16, 17], where state-level use of research in policymaking and funding of evidence-based treatments has declined in recent years in the USA [18]. Effective translation of research into policy at this level is critical, as states represent the primary funders of behavioral health services in the USA, and thus exercise tremendous influence over the standards and implementation requirements of behavioral health programs and practices [19].

The substantial time lag between when research is published and the uptake of findings into policy is well known [20]. This lag is generally attributed to the misaligned incentives between researchers (narrow view, long timeline) and policymakers (broad view, short timeline) [11], the lack of (general) policymaker capacity to independently gather and apply scientific findings to policy [21], and political influences on what knowledge to prioritize or consider [22]. Given this, long-term and mutually trusting relationship between researchers and policymakers is the most cited facilitator of the use of evidence in policy. The need for organizations to play a skilled role in the translational of research evidence as part of a system ecology of translation is also noted by multiple IS and knowledge translation frameworks [23]. The Interactive Systems Framework is one example of a model that calls for competencies in the translation of evidence and facilitation of its use at the decision-making level [24].

Several papers have explored and begun to catalog the competencies necessary to effectively translate research evidence [25, 26], but little is known about their prevalence in the wider ecology of political decision-making. “Intermediary” is a commonly used term to describe organizations that bridge the worlds of research-based knowledge and real-world systems [14, 15]. In behavioral health, existing efforts to describe the competencies of these organizations have focused on their ability to select, adapt, and support the flexible implementation of programs [14, 15]. In contrast, little is known about the activities of organizations disseminating evidence with the aim of improving or influencing behavioral health policy, in particular [27–29]. In this article, we use the term evidence-to-policy intermediaries (EPIs) to refer to this role as part of or distinct from activities taken by other types of behavioral health intermediary organizations.

EPIs already play a critical role in the knowledge production-to-application ecology. A better understanding of how these organizations operate, their current capacities, and funding infrastructure is a step towards understanding how to support better evidence-informed policy with policymakers and community partners [30].

### Current study

The current study surveyed leaders of EPI organizations to identify common strategies used to integrate research evidence into behavioral health policy and system-level decision-making. The study sought to quantify the degree to which EPIs use any of seventeen specific strategies, identified in the knowledge translation literature. The authors were also interested in the perceptions of EPI leadership regarding whether the academic literature on evidence translation helps EPI organizations advance their translational work.

Information from this study will help behavioral health policy researchers understand how EPIs provide support and guidance to policymakers and healthcare systems. Findings may elevate the role intermediary organizations play in influencing policy while highlighting the areas where targeted capacity building may enable such organizations to avoid overspecialization in a particular set of skills in favor of developing a suite of tools with which to engage policymakers to address complex needs.

## Methods

### Sample population

The sample included leaders within evidence-to-policy intermediary (EPI) organizations operating in English-speaking countries including the USA, Canada, the UK, and Australia. To guide the initial search, we defined EPIs as organizations providing behavioral health information to system leaders and policymakers. We then searched for EPIs through Internet searches, via the Society for Implementation Research Collaboration listserv, and by word of mouth. Internet searches used combinations of the terms *evidence-based*, *policy*, *center*, *support*, *implementation*, *mental health*, and *behavioral health*. Organizations were included in the sample pool if descriptions of center activities included providing system leaders and policymakers with evidence syntheses, policy analyses, reports with policy/system recommendations, or stakeholder engagement activities for the purpose of informing policy. For eligible organizations, we sought contact information for individuals likely to be knowledgeable about policy-supporting activities within that organization. Individuals in leadership positions including executive leadership, program directors, and implementation specialists were intentionally selected. Conversely, when Internet searches returned information about specific individuals, additional searches were conducted to locate that individual’s academic or research “home base” to determine if that agency qualified as an EPI. Upon compiling the above list of organizations, up to two key leaders per center were identified based on Internet searches of the organization’s website. This approach yielded 83 individuals from 44 intermediary organizations. Snowball sampling identified leaders representing seven additional organizations, for a total of 51 organizations and 90 individuals. The majority of the centers identified may be characterized as “arms-length” intermediaries, which operate outside the realm of both national government infrastructure and existing service delivery systems [27].

### Data collection

Surveys were sent via email using the Qualtrics platform. Prospective participants were notified that each completed survey would result in a $5 donation from the research team to a U.S. national non-profit organization serving homeless youth. Recruited individuals indicated an interest in participating by clicking a web link in the recruitment email, which began the online consent process, followed by the survey. Prospective participants were contacted up to three times, with email follow-up occurring 1 week and 2 weeks after the initial survey distribution to individuals yet to complete the survey. The study was approved by the University of Washington Institutional Review Board.

#### Survey development

To generate a list of evidence-to-policy intermediary activities, the research team conducted a literature review and a review of conference presentations of the Academy Health Annual Conference on the Science of Dissemination and Implementation to inform search terms. The literature review followed the PRIMSA-SCr guidelines [31]. Search terms included combinations of *policy AND research use*, *evidence use*, *research dissemination*, and *knowledge brokering.* Databases used in this search included Web of Science, PubMed, and Academic Search Complete.

Database searches were conducted in August 2020 and returned 581 titles for review. To be included in the final papers, one of the authors reviewed all of the titles and, when needed, abstracts to select publications for further review. Articles that described specific strategies taken to increase the use of evidence in decision-making were included. Fifty titles met the search criteria predetermined by the authors. Of these, one [32] was particularly useful as a source of specific evidence translation strategies. Two authors (LA and SCW) reviewed the documents and coded unique strategies which were then reviewed until the authors achieved consensus. The third author reviewed and agreed with the final list of strategies prior to survey development. Seventeen evidence-to-policy strategies were identified and included in the survey. Three strategies with modestly stronger empirical support, as judged by the authors (e.g., tailored evidence synthesis, researcher brokering, design-focused policymaking), were selected for more in-depth questioning.

### Measures

The survey included three major sections: (1) questions regarding the funding portfolios supporting evidence-to-policy activities; (2) the degree to which intermediary organizations used strategies from the published literature in their evidence-to-policy work, perceived benefits from these strategies, and were willing to pay for training on these strategies; and (3) participant perceptions regarding the relevance of the field of IS to their organization’s work.

#### Funding portfolios

Participants were asked to report the proportion of their organization’s funding portfolio among six categories, including (1) national government research grants, (2) foundation research grants, (3) government research contracts, (4) government service contracts, (5) foundation service contracts, and (6) organizational fundraising and private sources of funding. The mean proportions of the funding portfolio were calculated for each category, along with the number of funding sources identified per organization.

#### Evidence-to-policy strategies

The 17 unique evidence translation strategies identified from the literature review were classified into four categories: (1) evidence synthesis, (2) evidence dissemination, (3) activities connecting researchers to decision-makers, and (4) capacity building and implementation support. One author drafted the initial survey questions, which were iteratively refined with each co-author until consensus on language was achieved.

Each category was converted into a scale, with the identified strategies serving as indicators within each scale. Inter-item consistency was moderate to high across the four scales, with Cronbach’s alpha (*α*) ranging from 0.67 to 0.85. However, due to low inter-item consistency within the fourth category scale (capacity building and implementation support), the *training policymakers to critically appraise research* item was dropped. The scale *α* subsequently increased to 0.72. The evidence synthesis scale consisted of four strategies, evidence dissemination included three strategies, the research connecting activities scale comprised six strategy indicators, and the capacity building scale included three strategies. Cronbach’s alpha scores were calculated for each scale. One indicator strategy, “Training policymakers to critically appraise research,” was dropped from the capacity building scale due to poor inter-item consistency.

Participants were asked to indicate how central each of the 17 strategies, classified into the four categories identified above, was to the organization’s mission. Responses were recorded via 5-point Likert scales, ranging from “1 = not at all” to “5 = very much” for each item. The evidence synthesis category (1) consisted of four items, with an example strategy item reading, “*Summarizing research from multiple studies, like a formal evidence review*.” The evidence dissemination Sect. (2) included three strategies, an example of which read, “*Conducting and providing a review of the research evidence following the request of a decision-maker or policymaker*.” The connecting researchers to decision-makers Sect. (3) included seven items, an example of which read, “*Collaborating with traditionally marginalized and non-traditional partners to conduct research or translate evidence*.” Finally, the capacity building and implementation support Sect. (4) included four items to be rated, an example of which read, “*Facilitating co-design of new programs, systems, or processes using evidence*.”

Participants were then asked to estimate the likelihood that funders would support each of the four categories of strategies identified above. Responses were coded via 5-point Likert scales ranging from “1 = not at all” to “5 = very likely.”

To obtain a greater depth of information about evidence-to-policy strategies, while keeping the survey as brief as possible, the authors selected three evidence translation strategies for additional examination. Strategies were chosen based on the judgment of the authors (two of whom are active researchers in behavioral health policy and dissemination) regarding strategies that are more well-represented in the research literature. These included (1) request-driven evidence reviews, (2) connecting researchers to policymakers, and (3) structured policy and program design. Participants were provided a detailed paragraph defining each of these activities and asked to rate the degree to which (i) their organization conducted the activity regularly, (ii) the method was highly effective at influencing policymaking, and (iii) the participant was interested in learning more about that method. Responses were coded via 5-point Likert scales ranging from “1 = strongly disagree” to “5 = strongly agree.” Participants were additionally asked to estimate how much money their organization would be willing to pay to have a staff member attend a training on the method, ranging from $0 to $500. Willingness to pay for training served as an indicator of how much value the respondent placed on the skill set.

#### Perceptions of implementation science (IS)

Four questions assessed participants’ perceptions of the field of IS on a 0–100 scale. These included (1) how familiar are you with the field of IS with regard to your organization’s policy translation work, (2) how relevant is IS to your day-to-day policy translation work, (3) how actionable are the research findings from the field of IS to your policy translation work, and (4) how relevant is IS to your policy translation work.

#### Qualitative items

Respondents were asked to provide additional comments and feedback regarding the three in-depth evidence-to-policy strategies (request-driven evidence reviews, connecting research relationships, and structured policy and program design): *Please provide any additional thoughts or comments related to [strategy]*. In the IS use section, participants were asked to provide an open-ended response to the question: *What aspect(s) IS has/have been most useful to your practical work in influencing policymaking?*

### Analytic strategy

#### Quantitative analysis

Descriptive statistics, scales, significance tests, and internal consistency statistics were calculated using R. Independent sample *t*-tests and chi-square tests were calculated to compare the results between U.S. and international-based organizations, as well as university-based versus non-university-based organizations.

We used an open coding, inductive content analysis to extract themes from qualitative data [33]. Two authors (X and X) examined the qualitative responses separately to compare codes and organizing themes. The authors discussed coding until they reached a consensus. The qualitative coding was then shared with the third author who reviewed and provided feedback on the codes. Extracted items were discussed with co-authors and iteratively refined until consensus was met regarding how to describe each unique code and theme.

## Results

### Participants

Thirty-one participants from 27 agencies responded to the survey (participant response rate = 37%; agency response rate = 53%). Half of all respondents (52%) represented directors or executive leadership of their agency. Nearly one-quarter (23%) were employed as managers or program directors, and one-quarter (26%) served as program officers, implementation specialists, or professors.

### EPI characteristics

Representation was evenly split between EPIs based at universities (48%) and those without a university affiliation (52%). More than two-thirds (71%) of agencies were based in the USA, with additional organizations representing Canada (13%), Australia (10%), and the UK (7%). Seven organizations (23%) identified as research centers, four (13%) identified as training or best practices support centers, and three agencies (10%) served as consultation and/or information management support centers. Two EPIs (6%) were identified as both research and training support centers, while four organizations (13%) served as both training and consultation support center. Five agencies (16%) identified as a combination of research, training, and consultation centers, while six EPIs (19%) self-identified outside of these categories (e.g., conveners of policymakers).

### Agency funding

Funding mechanisms were diverse across EPIs. On average, government service contracts made up the largest share (29%) of EPI funding portfolios, followed by private fundraising (19%) and national government research grants (18%). Foundation research grants (7%) were the least frequently cited sources of funding. While funding was diverse between organizations (Table 1), EPIs tended to rely heavily on one or two funding sources for their operations. Overall, EPIs were supported by an average of 2.2 funding sources, with up to four sources reported by a small handful of agencies.

**Table 1:** Funding resources

	Full sample (*n* = 31), mean	University-based (*n* = 15)^a^, mean	Outside a university (*n* = 16), mean	*χ*^2^ (*p*-value)	USA (*n* = 21)^a^, mean	International (*n* = 10), mean	*χ*^2^ (*p*-value)
**Funding portfolio (%)**							
National government research grants	17.9 (25.7)	27.3 (28.7)	9.1 (19.5)	9.94 (0.002)**	21.7 (28.3)	10.0 (17.6)	4.29 (0.04)*
Foundation research grants	6.9 (16.0)	8.7 (18.4)	5.0 (13.3)	0.57 (0.45)	9.5 (18.9)	2.0 (6.3)	3.90 (0.05)*
Government research contracts	10.3 (23.7)	15.3 (29.9)	5.6 (15.5)	4.04 (0.04)*	6.9 (21.6)	17.5 (27.4)	4.30 (0.04)*
Government service contracts	29.0 (35.7)	27.2 (35.1)	30.6 (37.3)	0.14 (0.71)	35.0 (39.1)	16.3 (24.3)	8.21 (0.004)**
Foundation service contracts	14.9 (25.8)	9.8 (20.8)	20.0 (29.8)	3.34 (0.07)	11.5 (19.9)	21.7 (35.1)	3.06 (0.08)
Private sources; fundraising	18.7 (33.9)	11.7 (26.7)	25.3 (39.1)	5.26 (0.02)*	16.9 (32.7)	22.5 (38.0)	0.69 (0.41)
				*t* (95% CI)			*t* (95% CI)
**Number of funding sources**	2.2 (0.97)	2.47 (0.92)	1.88 (0.96)	1.76 (− 0.10, 1.28)	2.24 (0.89)	2.0 (1.15)	0.58 (− 0.65, 1.12)
**Likelihood funders will support activities (1–5)**							
Evidence synthesis	3.13 (1.31)	2.40 (1.30)	3.81 (0.91)	− 3.49 (− 2.25, − 0.58)**	3.00 (1.41)	3.33 (1.12)	− 0.87 (− 1.35, 0.55)
Evidence dissemination	3.65 (1.17)	3.47 (1.19)	3.81 (1.17)	− 0.82 (− 1.21, 0.52)	3.67 (1.06)	3.44 (1.42)	0.13 (− 1.02, 1.16)
Evidence connecting	2.87 (1.31)	2.53 (1.36)	3.19 (1.22)	− 1.41 (− 1.61, 0.30)	2.62 (1.28)	3.33 (1.32)	− 1.60 (− 1.81, 0.25)
Capacity-building and implementation support	3.77 (0.96)	3.73 (1.03)	3.81 (0.91)	− 0.23 (− 0.80, 0.64)	3.71 (1.01)	3.89 (0.93)	− 0.53 (− 0.92, 0.55)

^a^Reference group

^*^p < 0.05

^**^p < 0.01

While national government research grants were the third most frequent source of revenue overall, they were present in the portfolios of university-based agencies at three times the rate of non-university agencies (27.3% vs. 9.1%), a statistically significant finding (*p* < 0.01). Non-university-based agencies reported more government and foundation service contracts than university-based centers, but this did not reach statistical significance. Non-U.S.-based organizations were funded by foundation service contracts at roughly double the rate of U.S.-based organizations (21.7% vs. 11.5%) and had a higher proportion of government research contracts (17.5% vs. 6.9%, *p* = 0.04). Private fundraising made up a greater share of non-U.S. organization budgets as well (22.5% vs. 16.9%). Meanwhile, university-based (27.3 vs. 9.1, *p* < 0.01) and U.S.-based (21.7% vs. 10.0%, *p* = 0.04) organizations were more likely to be supported by national government research grants. U.S. agencies were more likely to be funded by non-national government service contracts (35.0% vs. 16.3%, *p* < 0.01). Foundation research grants, while comprising relatively small proportions of EPI budgets overall, made up a higher percentage of U.S.-based organization budgets (9.5%) than non-U.S. organizations (2.0%, *p* = 0.05).

### Likelihood of funding for EPI strategies

Participants were asked to estimate the degree to which funders would financially support each of the four evidence use scales. Capacity-building and implementation support had the highest score (mean 3.77 out of 5), although none of the categories exceeded “likely.” EPIs based outside of universities had higher mean scores for each category, but only one comparison reached statistical significance: compared to their university-based counterparts (2.40), non-university EPIs were more likely to believe that activities in the evidence synthesis category could be funded (3.81, *p* < 0.01) (see Table 1).

### Evidence-to-policy items and scales

We observed moderate variation (range of 1.79, 5-point scale) in the reported frequency of use among the individual items. Four items scored at 4.0 or above (“very much—this is a core activity of ours”). These core strategies included (1) direct service improvement activities, *m* = 4.19; (2) facilitating co-design of new programs, systems, or processes, *m* = 4.03; (3) training and supporting leadership planning skills to acquire and implement new knowledge, *m* = 4.03; and (4) conducting interactive workshops designed for specific audiences to facilitate the use of evidence in planning, *m* = 4.00 (see Table 2).

**Table 2:** Evidence translation strategies

	Full sample (*n* = 31)		University-based (*n* = 15)^b^, mean (SD)	Outside a university (*n* = 16), mean (SD)	*t* (95% CI)	USA (*n* = 21)^b^, mean (SD)	International (*n* = 10), mean (SD)	*t* (95% CI)
	Mean (SD)	Core activity, *n* (%)^a^						
**Evidence synthesis activities (score: 2.92, *α* = 0.67)**					− 0.01 (− 0.84, 0.83)			0.29 (− 0.89, 1.17)
Summarizing single studies	3.10 (1.56)	13 (41.9)	2.93 (1.67)	3.25 (1.48)	− 0.56 (− 1.48, 0.85)	3.24 (1.45)	2.78 (1.92)	0.67 (− 0.96, 1.84)
Summarizing multiple studies	3.52 (1.55)	18 (58.1)	3.67 (1.54)	3.38 (1.59)	0.52 (− 0.86, 1.44)	3.62 (1.50)	3.33 (1.80)	0.51 (− 1.02, 1.66)
Developing evidence reviews	2.68 (1.72)	11 (35.5)	2.93 (1.83)	2.44 (1.63)	0.79 (− 0.78, 1.77)	2.57 (1.66)	3.00 (2.00)	− 0.47 (− 1.83, 1.17)
Maintaining research database	2.40 (1.43)	7 (23.3)	2.13 (1.51)	2.67 (1.35)	− 1.02 (− 1.60, 0.54)	2.45 (1.43)	2.33 (1.58)	0.26 (− 1.05, 1.35)
**Evidence dissemination activities (score: 3.62, *α* = 0.79)**					0.10 (− 0.81, 0.89)			0.82 (− 0.71, 1.56)
Distributing research summaries	3.55 (1.29)	16 (51.6)	3.60 (1.18)	3.50 (1.41)	0.21 (− 0.86, 1.06)	3.52 (1.25)	3.67 (1.50)	− 0.14 (− 1.20, 1.04)
Hosting didactic webinars	3.71 (1.35)	17 (54.8)	3.67 (1.29)	3.75 (1.44)	− 0.17 (− 1.09, 0.92)	3.90 (1.18)	3.33 (1.73)	1.05 (− 0.64, 1.85)
Conducting research reviews for policymakers	3.61 (1.43)	20 (64.5)	3.67 (1.29)	3.56 (1.59)	0.20 (− 0.96, 1.17)	3.86 (1.28)	3.22 (1.72)	1.27 (− 0.52, 2.03)
**Evidence connecting activities (score: 3.55, *α* = 0.85)**					− 0.08 (− 0.78, 0.72)			− 1.04 (− 1.12, 0.37)
Maintaining active system-level partnerships	3.47 (0.90)	24 (80.0)	4.53 (0.99)	4.40 (0.83)	0.40 (− 0.55, 0.82)	4.30 (1.03)	4.89 (0.33)	− 1.88 (− 1.05, 0.05)
Collaborating with traditionally marginalized and non-traditional partners	2.84 (1.39)	10 (32.3)	2.87 (1.46)	2.81 (1.38)	0.11 (− 0.99, 1.10)	2.90 (1.48)	2.56 (1.24)	0.40 (− 0.86, 1.27)
Participating in policy advisory positions	3.68 (1.22)	19 (61.3)	3.67 (1.18)	3.69 (1.30)	− 0.05 (− 0.93, 0.89)	3.67 (1.24)	3.67 (1.32)	− 0.07 (− 1.04, 0.98)
Conducting interactive workshops	4.00 (1.29)	22 (71.0)	4.00 (1.00)	4.00 (1.55)	0.00 (− 0.96, 0.96)	3.76 (1.37)	4.44 (1.01)	− 1.72 (− 1.62, 0.15)
Hosting informational forums with policymakers	3.23 (1.43)	15 (48.4)	3.00 (1.25)	3.44 (1.59)	− 0.85 (− 1.49, 0.61)	3.00 (1.52)	3.67 (1.22)	− 1.42 (− 1.72, 0.32)
Facilitating one on one conversations between researchers and policymakers	3.06 (1.61)	13 (41.9)	3.13 (1.73)	3.00 (1.55)	0.23 (− 1.08, 1.34)	2.90 (1.61)	3.44 (1.74)	− 0.79 (− 1.82, 0.83)
**Capacity-building and implementation support (score: 4.09, *α* = 0.72)**					− 1.43 (− 1.25, 0.22)			− 1.51 (− 1.22, 0.19)
Direct service improvement activities to support process implementation	4.19 (1.25)	23 (74.2)	4.00 (1.51)	4.38 (0.96)	− 0.82 (− 1.32, 0.57)	4.19 (1.25)	4.11 (1.36)	− 0.02 (− 1.06, 1.04)
Facilitating co-design of new programs, systems, or processes	4.03 (1.33)	22 (71.0)	3.73 (1.49)	4.31 (1.14)	− 1.21 (− 1.56, 0.40)	3.86 (1.42)	4.33 (1.12)	− 1.18 (− 1.50, 0.41)
Training and supporting leadership planning skills to acquire and implement new knowledge	4.03 (1.17)	23 (74.2)	3.73 (1.22)	4.31 (1.08)	− 1.40 (− 1.43, 0.27)	3.71 (1.27)	4.67 (0.50)	− 3.11 (− 1.63, − 0.34)**
**Miscellaneous**								
Training policymakers to critically appraise research	2.87 (1.48)	9 (29.0)	2.73 (1.39)	3.00 (1.59)	− 0.50 (− 1.36,0.83)	2.71 (1.19)	3.00 (2.00)	− 0.71 (− 1.97, 0.99)

^a^Share of participants responding “moderately” or “very much”

^b^Reference group

^**^p < 0.01

Conversely, four strategies scored below a threshold of 3.0 (“somewhat—we do this a little”). These least employed strategies by EPIs were (1) maintaining a database of research-based information, *m* = 2.40; (2) developing evidence reviews using formal critical appraisal methods, *m* = 2.68; (3) collaborating with traditionally marginalized and non-traditional partners to conduct research or translate evidence, *m* = 2.84; and (4) training and coaching policymakers to do a critical appraisal of the research literature, *m* = 2.87.

We observed a large range in correlations among the four evidence use scales (e.g., *r* =  − 0.02–0.70), suggesting it was uncommon for organizations to be involved in all four types of evidence-to-policy translation. We observed nearly zero correlation (− 0.02) between the capacity building and evidence synthesis scales and between the capacity building and evidence dissemination categories (0.09). This suggests that organizations involved in predominately evidence synthesis and dissemination activities (e.g., developing and sharing evidence reviews) are highly unlikely to be engaged in capacity-building activities (e.g., codesigning policies, working with marginalized populations). We observed a moderate correlation (0.37) between the connecting researchers to policymakers and evidence synthesis scales, as well as a moderate correlation between the capacity building and connecting researchers to policymakers scales (0.34). Evidence synthesis and evidence dissemination were also moderately correlated (0.64). The strongest correlation was observed between evidence dissemination and connecting researchers to policymakers (0.70) (Table 3).

**Table 3:** Scale scores

	**Scale intercorrelations corrected for attenuation**			
	**1**	**2**	**3**	**4**
**1. Evidence synthesis**	–			
**2. Evidence dissemination**	0.64	–		
**3. Evidence connecting**	0.37	0.70	–	
**4. Capacity-building**	− 0.02	0.09	0.34	–

### Perceived value of strategies

Non-university-based organizations viewed request-driven evidence reviews (RDERS) as more effective at influencing policy- than university-based organizations (3.94 vs. 3.40, *p* < 0.05). However, university-based EPIs expressed a greater willingness to pay for training in request-driven evidence reviews (RDER) ($262 vs. $128, *p* < 0.05). U.S. organizations expressed a stronger interest in learning about RDERs (mean 3.74 vs. 2.17, *p* < 0.05) compared to their international counterparts. As will be reviewed below, the qualitative results suggest that this likely due to non-U.S. organizations having more expertise in conducting RDERs than U.S. organizations. Other comparisons did not reveal meaningfully or statistically significant differences between U.S. and non-U.S. or university and non-university organizations (see Table 4).

**Table 4:** Perceptions of implementation science

	Full sample (*n* = 31), mean (SD)	University-based (*n* = 15)^a^, mean (SD)	Outside a university (*n* = 16), mean (SD)	*t* (95% CI)	USA (*n* = 21)^a^, mean (SD)	International (*n* = 10), mean (SD)	*t* (95% CI)
**Request-driven evidence reviews (1–5)**							
Our organization uses this method regularly	3.00 (1.51)	3.33 (1.45)	2.69 (1.54)	1.20 (− 0.45, 1.74)	2.95 (1.40)	3.33 (1.73)	− 0.58 (− 1.79, 1.03)
This method is highly effective at influencing behavioral health policymaking	3.68 (0.70)	3.40 (0.63)	3.94 (0.68)	− 2.28 (− 1.08, − 0.06)*	3.71 (0.64)	3.56 (0.88)	0.49 (− 0.55, 0.87)
I am interested in learning more about this method	3.38 (1.20)	3.36 (1.01)	3.42 (1.44)	− 0.12 (− 1.10, 0.98)	3.74 (0.99)	2.17 (1.17)	2.97 (0.33, 2.81)*
My organization would pay $____ to attend or have a staff member attend a workshop about this topic ($0–500)	203.6 (168.4)	262.1 (196.5)	127.5 (80.3)	2.24 (7.58, 261.58)*	222.6 (184.6)	144.0 (66.0)	1.44 (− 38.09, 195.20)
**Connecting research relationships (1–5)**							
Our organization uses this method regularly	3.32 (1.45)	3.67 (1.45)	3.00 (1.41)	1.30 (− 0.39, 1.72)	3.48 (1.36)	3.11 (1.69)	0.57 (− 1.02, 1.75)
This method is highly effective at influencing behavioral health policymaking	3.71 (0.97)	3.67 (0.90)	3.75 (1.06)	− 0.24 (− 0.81, 0.64)	3.86 (1.01)	3.44 (0.88)	1.12 (− 0.36, 1.19)
I am interested in learning more about this method	2.92 (1.32)	2.77 (0.93)	3.09 (1.70)	− 0.56 (− 1.54, 0.90)	3.24 (1.35)	2.17 (0.98)	2.06 (− 0.06, 2.19)
My organization would pay $____ to attend or have a staff member attend a workshop about this topic ($0–500)	166.8 (191.0)	231.0 (231.0)	113.2 (138.4)	1.41 (− 60.63, 296.13)	187.9 (208.8)	95.0 (94.2)	1.41 (− 46.98, 232.74)
**Structured policy and program design (1–5)**							
Our organization uses this method regularly	3.29 (1.42)	3.01 (1.58)	3.50 (1.26)	− 0.84 (− 1.49, 0.63)	3.24 (1.41)	3.44 (1.59)	− 0.34 (− 1.52, 1.11)
This method is highly effective at influencing behavioral health policymaking	3.80 (0.89)	3.73 (0.59)	3.87 (1.13)	− 0.41 (− 0.82, 0.55)	3.85 (0.99)	3.78 (0.67)	0.23 (− 0.58, 0.72)
I am interested in learning more about this method	3.81 (0.94)	3.79 (1.05)	3.83 (0.83)	− 0.13 (− 0.81, 0.72)	3.74 (0.99)	4.17 (0.75)	− 1.12 (− 1.27, 0.41)
My organization would pay $____ to attend or have a staff member attend a workshop about this topic ($0–500)	256.4 (209.6)	280.0 (188.9)	232.7 (236.3)	0.49 (− 154.38, 248.98)	231.7 (232.9)	330.4 (98.5)	− 1.32 (− 256.41, 58.95)
**Perceptions of implementation science (IS) (0–100)**							
Familiarity with IS regarding the organization’s policy translation work	81.8 (21.9)	76.7 (28.6)	86.6 (11.9)	− 1.25 (− 26.64, 6.72)	79.3 (20.4)	87.8 (26.4)	− 0.86 (− 29.84, 12.95)
Relevance of IS to day-to-day policy translation work	83.6 (19.6)	85.4 (17.2)	81.9 (22.0)	0.49 (− 11.02, 17.94)	79.8 (21.2)	91.8 (13.9)	− 1.83 (− 25.51, 1.57)
IS research findings are actionable to policy translation work	62.5 (25.1)	64.4 (25.4)	60.9 (25.6)	0.37 (− 15.71, 22.54)	59.0 (25.0)	67.1 (23.8)	− 0.81 (− 29.73, 13.48)
Relevance of IS to overall policy translation work	82.6 (22.9)	88.1 (17.5)	77.9 (26.3)	1.26 (− 6.39, 26.78)	77.3 (25.1)	94.4 (9.0)	− 2.69 (− 30.04, − 4.04)*

^a^*t*-test reference group

^*^p < 0.05

### Perceptions of implementation science

EPIs expressed generally positive views towards the discipline of IS. Out of a possible score of 100, three items scored above 80: (1) familiarity with IS regarding the organization’s policy translation work, (2) relevance of IS to day-to-day policy translation activities, and (3) relevance of IS to overall policy translation work. Non-U.S. organizations held more positive views of IS compared to U.S. organizations on all four indicators. The lowest scored item in this Sect. (62.5/100) was the prompt *IS research findings are actionable to policy translation work* (Table 4).

### Qualitative results

#### Request-Driven Evidence Reviews (RDERs)

Qualitative analyses were viewed as exploratory prompts to guide the development of hypotheses for future studies about how evidence-to-policy strategies are currently used and viewed among real-world EPIs. As the prompts for the qualitative questions were neutral (e.g., please add any additional thoughts), we view comments about the value of these strategies or concerns to be meaningful signifiers of respondents’ experiences in the field. Eleven participants responded to the open-ended question about the value of RDERs. The timing of incorporating RDERs into an EPI strategy was a prominent theme among respondents. Three participants viewed RDERs as a valuable strategy for influencing policymaking in the immediate term, noting that RDERs represented a core function of their agency’s activities. Two participants noted that they were not currently conducting RDERs but thought they might adopt this strategy in the future.

Funding was a concern raised by two participants, who noted that both current budgets and potential funders constrain the ability or willingness to center RDERs among an EPI’s core activities. Additionally, one respondent expressed a concern that RDERs are only one piece of a complex policymaking puzzle, limiting their independent effectiveness. Another respondent suggested that the impact of RDERs may only be relevant to policymakers who “have already bought into the idea of evidence-informed decision making and that are part of the process of the review.”

Two participants expressed interest in comparing and improving the methods of producing RDERs, such as “staying on top of improvements and changes in these methods,” and mentioned that they “would be interested in learning how others field policymaker [RDER] requests in an efficient and rigorous manner.” A desire to meet with other organizations to share best practices and contrast RDER methods was also noted.

#### Connecting researchers to policymakers

Ten participants provided comments about the connecting researchers to policymakers strategies. Multiple agencies reported this strategy as a core element of their work (*n* = 4), with one participant noting, “This is a key area for our business, as our customers (government and health and human services agencies) frequently find it difficult to communicate with academic researchers, but are motivated to understand and incorporate research findings into policy and practice.” Meanwhile, others noted that such connecting activities are engaged informally or might be something that “happens organically at events we organize on specific topics.”

Responses suggested that researcher-policymaking connections occurred because they were required by funders or happened unpredictably. One organization reported such activities being a requirement of their EPI’s funding source, while another noted the strategy was utilized based on the relational networks and connections to researchers, policy designers, and implementation specialists cultivated by employees of the organization. Two participants noted that existing relationships with policymakers were also beneficial for informing research ideas.

A concern about researcher connecting activities included the risk of bias. One participant noted the potential for politicization of such work. Another response highlighted the subjectivity of relational networks, which may complicate attempts to establish a centralized list of stakeholders from which to connect researchers and policymakers. An additional comment expressed a caveat around the strategic effectiveness of connecting activities, noting, “making these connections is a good step, but [I am] not sure if it’s highly effective in the fabric of complex decision-making.”

#### Structured policy and program design

Seven participants commented on structured policy and program design. Two respondents noted that these activities were woven into their existing methods and organizational practices. However, they reported that these activities lack formal structure. One respondent commented on their efforts to translate research evidence into policy recommendations, “it would have been helpful to actually learn from policymakers what was most helpful to them.”

Respondents expressed interest in learning more about how other similarly situated centers were attempting policy design. One respondent noted, “I would be very interested in how to influence policy in a more structured, systematic way and relying less on the informal interactions.” Another participant noted a desire to compare their use of this strategy with that of other stakeholders.

#### Practical use of implementation science in policymaking

Seventeen participants (55.8%) provided comments regarding the most useful facets of IS to their practical work influencing policymaking. A core theme emerging from the responses was the strength of IS frameworks for facilitating knowledge translation. One respondent wrote, “implementation science…allows for a more thoughtful and successful translation of research into practice.” Another commented, “findings of implementation science have been helpful to us in prioritizing our [policy] recommendations.”

Additional comments highlighted the usefulness of systems thinking and systematic approaches to knowledge translation (*n* = 5), strategies for effective program implementation (*n* = 3), practical implementation studies and tools (*n* = 5), and the inclusion of multiple voices in the policymaking space (*n* = 3). In contrast, one participant saw IS as not sufficiently addressing real-world conditions, contending “research sometimes focuses more on describing characteristics of successful implementation efforts that are not necessarily highly changeable.”

## Discussion

EPIs play a role distinct from other types of behavioral health intermediary organizations described in the field. This is particularly true regarding the methods used to narrow the evidence-to-policy gap while ensuring the application of evidence is responsive to local political conditions. We aimed to illuminate the EPI landscape with a snapshot of activities undertaken to influence behavioral health policy. A key finding is that EPI organizations did not engage in activities across the range of translation categories. Instead, EPIs tended to specialize in either synthesis/dissemination activities or system improvement/capacity-building activities.

Few organizations offer the range of research translation services envisioned by implementation models, such as the Interactive Systems Framework (ISF) [24]. The ISF, for example, argues for the need for three “systems” that “optimally work together for successful dissemination and implementation of prevention innovations” (pp. 178). An example of an effort with overlapping capacities is the Rapid Synthesis and Translation Process (RSTP) supported by the Division of Violence Prevention at the Centers for Disease Control and Prevention. The RSTP process is conceptualized as a synthesis activity, but with significant stakeholder (practitioner, system level partners, and policy) engagement. System practitioners select the topics chosen for research synthesis as well as the presentation of findings (actionable, concrete, brief). Service leaders and practitioners may then apply review findings to their work (e.g., worksheets, tools, and resources used to improve practice) [34].

The two least endorsed EPI activities in our study were evidence synthesis and collaborating with traditionally marginalized communities. Most EPIs in our survey appeared to be working in service improvement and capacity building and likely had a primary audience of policymakers and service providers. This has at least two implications for policy development. Increasingly, the field of health services research is concerned with the need to attend to equity in the processes of conducting research and translating research findings. The National Academy of Medicine, for example, recently published a white paper revisiting existing recommendations for improvement and calling for more community engagement and partnership [35]. As argued by the paper, community and patient engagement is critical because ownership in the policymaking process engenders better ideas for service improvement and trust in the healthcare system. EPIs can play a critical role for policymakers by engaging community into policy formation and implementation [36], as well as supporting the integration of information arising from the research evidence base with community perspectives [37, 38]. Integrating this information can be facilitated by an increased capacity to conduct synthesis activities in which core principles and elements can be extracted from the research literature and applied to fit local needs and priorities [39].

The need for interconnected supports and the limited value of any single evidence-to-policy activity was echoed by comments in the qualitative portion of the study. Respondents generally viewed responsive evidence reviews positively but felt that their impact was contingent on external conditions. Similarly, respondents felt that connecting researchers to policymakers had bidirectional benefits but could be problematic if used to support political positions. “Arms-length” EPIs may be more successfully positioned to maintain intellectual independence than other types of embedded or informal intermediary relationships [27].

Funding availability is a key factor affecting which activities EPIs undertake. A topic not often addressed in the IS literature is the real-world market for behavioral health intermediaries in general, and policy intermediaries in particular. Franks and Bory’s [40] review of intermediaries found most centers began as service training entities (a service most governments recognize as valuable), gradually building long-term relationships with government organizations that provided a pathway for more influence on policy formation. In the present study, most EPIs (73.5%) secured funding from more than one source, with one-third (32.3%) of agencies incorporating three or more of the six funding sources within their portfolio. Notably, EPI organizations and activities are unlikely to be funded by national government research grants. This is consistent with research demonstrating the low frequency of NIH grants focused on behavioral health policy [41]. EPI activities are more often supported by government service grants and contracts, with some support from foundations. We observed a meaningful difference between U.S. and non-U.S. funding sources, with more private fundraising and government contracts (non-competitive support) outside of the U.S. This is consistent with the growing literature on intermediaries operating outside the U.S., which face acute challenges of scarce or unpredictable funding [42]. Optimism among respondents for funding any of the EPI activities was low, particularly for evidence synthesis, suggesting there may be more difficulty funding activities that support policy formation compared to service improvement. This may be because governments and policymakers feel urgency around action and have a lower appetite for funding planning activities [43].

Overall, the findings provide a reason to be optimistic about the state of the “synthesis” and “capacity” building systems for behavioral health policymaking. We found that among our sample, the frequency of EPI activities was reasonably high. This provides a solid foundation for building a community of practice among entities doing IS-informed work. The high endorsement of IS in general suggests these organizations are active users of the research literature and are likely to adopt methods found to be effective for evidence-informed policymaking. However, respondents felt that IS could provide more meaningful guidance in this specific practice area.

Finally, there is a business case to be made for EPIs to establish a diverse portfolio of evidence translation strategies. For example, both university-based and non-university agencies were willing to pay for training. EPIs incorporating these methods may open an untapped revenue stream that diversifies their funding portfolio while expanding their sphere of influence simultaneously.

## Limitations

The study is limited to agencies identified through Internet searches, listserv dissemination, and snowball sampling. The sample almost certainly underrepresents organizations doing similar work. A post hoc estimate of respondents suggests about 70–80% came from direct requests following an Internet search or because the authors were already aware of the organization, and consequently, the results overrepresent “arms-length” intermediaries that are more likely to be connected to implementation science or health service research. We recognize that the definition of EPI is porous and future research might examine how these types of organizations differ and overlap with researcher-practice partnerships, university-community partnerships, and other similarly oriented efforts. Furthermore, as respondents exclusively came from Anglophone countries, we cannot generalize findings to the strategies or activities undertaken outside of these political environments. Other types of organizations may have different perspectives regarding what works to successfully integrate evidence-based information into policy and may have differing levels of interest in being part of a practice community.

## Conclusion

EPIs play a critical role in the knowledge production-to-application ecology of behavioral health policymaking. However, while EPIs are employing the research translation strategies found in the IS literature, such use remains highly specialized, as do the funding portfolios of these agencies. Polyspecialization is likely needed to harness translational impact. A particular need exists for EPIs to work with communities affected by policies, especially around behavioral health and social welfare. Additionally, EPIs use IS to translate research into policy; however, the perceived actionability of IS for agencies’ own policy translation methods was limited. Finally, there is a business case to be made for translating IS methods that could be income-generating for training organizations seeking to build the capacity of EPIs.

## Supplementary Information

**Additional file 1.**

## Data Availability

The datasets generated and/or analyzed during the current study are available in the Open Science Framework repository, https://doi.org/10.17605/OSF.IO/Z683E.

## Abbreviations

IS: Implementation science
EPI: Evidence-to-policy intermediary
U.S.: United States
RDER: Request-driven evidence review
ISF: Interactive systems framework
RSTP: Rapid synthesis and translation process
